# Metabolomics approach reveals effects of antihypertensives and lipid-lowering drugs on the human metabolism

**DOI:** 10.1007/s10654-014-9910-7

**Published:** 2014-05-10

**Authors:** Elisabeth Altmaier, Gisela Fobo, Margit Heier, Barbara Thorand, Christine Meisinger, Werner Römisch-Margl, Melanie Waldenberger, Christian Gieger, Thomas Illig, Jerzy Adamski, Karsten Suhre, Gabi Kastenmüller

**Affiliations:** 1 Institute of Bioinformatics and Systems Biology, Helmholtz Zentrum München, German Research Center for Environmental Health, Ingolstädter Landstr. 1, 85764 Neuherberg, Germany; 2 Institute of Epidemiology II, Helmholtz Zentrum München, German Research Center for Environmental Health, Ingolstädter Landstr. 1, 85764 Neuherberg, Germany; 3 Research Unit of Molecular Epidemiology, Helmholtz Zentrum München, German Research Center for Environmental Health, Ingolstädter Landstr. 1, 85764 Neuherberg, Germany; 4 Institute of Genetic Epidemiology, Helmholtz Zentrum München, German Research Center for Environmental Health, Ingolstädter Landstr. 1, 85764 Neuherberg, Germany; 5 Hannover Unified Biobank, Hannover Medical School, Carl-Neuberg-Str. 1, 30625 Hannover, Germany; 6 Institute of Experimental Genetics, Genome Analysis Center, Helmholtz Zentrum München, German Research Center for Environmental Health, Ingolstädter Landstr. 1, 85764 Neuherberg, Germany; 7 Institute of Experimental Genetics, Life and Food Science Center Weihenstephan, Technische Universität München, 85354 Freising, Germany; 8 Department of Physiology and Biophysics, Weill Cornell Medical College in Qatar, Education City, Qatar Foundation, PO Box 24144, Doha, State of Qatar

**Keywords:** Beta-blockers, Angiotensin-converting enzyme inhibitors, Diuretics, Statins, Fibrates, Metabolomics

## Abstract

The mechanism of antihypertensive and lipid-lowering drugs on the human organism is still not fully understood. New insights on the drugs’ action can be provided by a metabolomics-driven approach, which offers a detailed view of the physiological state of an organism. Here, we report a metabolome-wide association study with 295 metabolites in human serum from 1,762 participants of the KORA F4 (Cooperative Health Research in the Region of Augsburg) study population. Our intent was to find variations of metabolite concentrations related to the intake of various drug classes and—based on the associations found—to generate new hypotheses about on-target as well as off-target effects of these drugs. In total, we found 41 significant associations for the drug classes investigated: For beta-blockers (11 associations), angiotensin-converting enzyme (ACE) inhibitors (four assoc.), diuretics (seven assoc.), statins (ten assoc.), and fibrates (nine assoc.) the top hits were pyroglutamine, phenylalanylphenylalanine, pseudouridine, 1-arachidonoylglycerophosphocholine, and 2-hydroxyisobutyrate, respectively. For beta-blockers we observed significant associations with metabolite concentrations that are indicative of drug side-effects, such as increased serotonin and decreased free fatty acid levels. Intake of ACE inhibitors and statins associated with metabolites that provide insight into the action of the drug itself on its target, such as an association of ACE inhibitors with des-Arg(9)-bradykinin and aspartylphenylalanine, a substrate and a product of the drug-inhibited ACE. The intake of statins which reduce blood cholesterol levels, resulted in changes in the concentration of metabolites of the biosynthesis as well as of the degradation of cholesterol. Fibrates showed the strongest association with 2-hydroxyisobutyrate which might be a breakdown product of fenofibrate and, thus, a possible marker for the degradation of this drug in the human organism. The analysis of diuretics showed a heterogeneous picture that is difficult to interpret. Taken together, our results provide a basis for a deeper functional understanding of the action and side-effects of antihypertensive and lipid-lowering drugs in the general population.

**Electronic supplementary material:**

The online version of this article (doi:10.1007/s10654-014-9910-7) contains supplementary material, which is available to authorized users.

## Introduction

Hypertension is a risk factor for the development of atherosclerosis and cardiovascular diseases as well as for renal failure. Obesity and hyperlipidemia increase the risk of cardiovascular disease as well, as well as of diabetes mellitus type 2, gout and cognitive decline, including Alzheimer’s disease. In addition to efforts to change life-style, antihypertensive drugs and lipid-lowering agents are prescribed. Due to the high prevalence of these risk factors, this medication ranks among the most frequently prescribed drugs in medical practice.

Beta-blockers are a heterogeneous class of antihypertensive drugs. Their main mode of action is the inhibition of noradrenaline and adrenaline β-adrenergic receptors, which diminishes the effect of the sympathetic nervous system on its target organ, especially on the smooth muscle tissue of the blood vessels and the heart. Most beta-blockers act on β_1_-, β_2_- and/or β_3_-adrenergic receptors, while some beta-blockers of the newer generation show an additional vasodilatory effect by, for example, blocking α_1_-adrenergic receptors [1–3]. Another important class of antihypertensive agents are angiotensin-I-converting enzyme (ACE) inhibitors. The ACE catalyzes the conversion of angiotensin-I to angiotensin-II which causes the muscles surrounding blood vessels to contract, thereby restricting the blood flow and increasing blood pressure. In addition, ACE cleaves and reduces the action of the vasodilator bradykinin and, thereby, increases the blood pressure. ACE inhibitors diminish the action of this enzyme and, thus, blood vessels dilate and blood pressure is reduced [1, 4]. A drug therapy with diuretics has long been one of the most important treatments of hypertension and heart failure. However, today, many diuretics have found their main role in areas outside of hypertension. Diuretics inhibit the reabsorption of sodium in the renal tubules and their ability to alter long-term sodium balance induces important hemodynamic changes that result in a reduction in peripheral resistance and a decrease in blood pressure [5–7].

Two important classes of lipid-lowering drugs are statins and fibrates. Statins lower the concentration of cholesterol in the blood by reducing the cholesterol biosynthesis. The rate-limiting enzyme of this biosynthesis is the 3-hydroxy-3-methyl-glutaryl-CoA reductase (HMG-CoA reductase; HMGCR), which is competitively inhibited by the statins [8, 9]. In contrast, fibrates decrease the blood levels of fatty acids and triglycerides. They activate the peroxisome proliferator-activated receptor (PPAR), mainly PPAR-α, which mediates the stimulation of the fatty acid β-oxidation [10–13].

In this study we used a pharmacometabolomic approach. Pharmacometabolomics aims to identify metabolic traits that offer insights into the intended as well as the unintentional effects of drugs on the human organism. The power of pharmacometabolomics was already shown in several studies [14–16]. One example is the study from Trupp et al. [14]. Examining the metabolic response to simvastatin treatment in 148 study participants they identified metabolites from multiple pathways not directly connected to the drug target which may explain the variation in therapeutic response.

For the identification of drug effects clinical drug testing is the standard approach. These trials are conducted under very controlled conditions and they are mainly focused on the objectives of the trials. The metabolomics approach for the analysis of medication effects in a big study population can provide a broader picture of the pathways affected by the respective drug by giving a snapshot of the physiological state of the organism. Using this kind of analysis, hypotheses about on-target as well as off-target effects can be generated, that reflect the drug action in the population and under everyday life conditions. A population based approach also allows the analysis of several drugs in the same study.

In this study, we used a mass spectrometry based non-targeted metabolomics approach to investigate the effect of antihypertensive and lipid-lowering agents. To this end, we analyzed the metabolic profiles of 1,762 participants from the KORA (Cooperative Health Research in the Region of Augsburg) study population for associations with the intake of these drugs (classification into cases/controls see Table 1).

**Table 1:** Characteristics of the population related to antihypertensives and lipid-lowering drugs

*a*	Intake of beta-blockers Mean (SD) or n (%)	
	Yes (n = 403)	No (n = 1,359)
Age	65.43 (7.45)	59.46 (8.66)
Gender		
Male	201 (11.4)	655 (37.2)
Female	202 (11.5)	704 (40.0)
HDL-cholesterol (mmol/l)	1.34 (0.35)	1.49 (0.38)
LDL-cholesterol (mmol/l)	3.39 (0.84)	3.67 (0.91)
Total cholesterol (mmol/l)	5.43 (0.95)	5.81 (1.01)
Triglycerides (mmol/l)	1.68 (1.0)	1.45 (1.05)
BMI kg/m^2^	30.13 (4.95)	27.58 (4.62)
Diabetes mellitus		
Yes	69 (3.9)	92 (5.2)
No	334 (19.0)	1,267 (71.9)
Hypertension		
Yes	347 (19.7)	308 (17.5)
No	56 (3.2)	1,051 (59.6)
Intake of ACE inhibitors		
Yes	139 (7.9)	143 (8.1)
No	264 (15.0)	1,216 (69.0)
Intake of diuretics		
Yes	219 (12.4)	156 (8.9)
No	184 (10.4)	1,203 (68.3)
Intake of statins		
Yes	144 (8.2)	133 (7.5)
No	259 (14.7)	1,226 (69.6)
Intake of fibrates		
Yes	5 (0.3)	6 (0.3)
No	398 (22.6)	1,353 (76.8)

*b*	Intake of ACE inhibitors Mean (SD) or n (%)	
	Yes (n = 282)	No (n = 1,480)
Age	65.97 (7.77)	59.84 (8.60)
Gender		
Male	165 (9.4)	691 (39.2)
Female	117 (6.6)	789 (44.8)
HDL-cholesterol (mmol/l)	1.34 (0.33)	1.48 (0.38)
LDL-cholesterol (mmol/l)	3.42 (0.89)	3.65 (0.90)
Total cholesterol (mmol/l)	5.47 (0.99)	5.77 (1.01)
Triglycerides (mmol/l)	1.69 (1.01)	1.47 (1.05)
BMI kg/m^2^	30.55 (5.03)	27.71 (4.63)
Diabetes mellitus		
Yes	70 (4.0)	91 (5.2)
No	212 (12.0)	1,389 (78.8)
Hypertension		
Yes	261 (14.8)	394 (22.4)
No	21 (1.2)	1,086 (61.6)
Intake of beta-blockers		
Yes	139 (7.9)	264 (15.0)
No	143 (8.1)	1,216 (69.0)
Intake of diuretics		
Yes	178 (10.1)	197 (11.2)
No	104 (5.9)	1,283 (72.8)
Intake of statins		
Yes	99 (5.6)	178 (10.1)
No	183 (10.4)	1,302 (73.9)
Intake of fibrates		
Yes	3 (0.2)	8 (0.5)
No	279 (15.8)	1,472 (83.5)

*c*	Intake of diuretics Mean (SD) or n (%)	
	Yes (n = 375)	No (n = 1,387)
Age	65.90 (7.44)	59.45 (8.59)
Gender		
Male	195 (11.1)	661 (37.5)
Female	180 (10.2)	726 (41.2)
HDL-cholesterol (mmol/l)	1.37 (0.33)	1.48 (0.38)
LDL-cholesterol (mmol/l)	3.40 (0.84)	4.67 (0.91)
Total cholesterol (mmol/l)	5.46 (0.99)	5.79 (1.01)
Triglycerides (mmol/l)	1.66 (0.98)	1.47 (1.05)
BMI kg/m^2^	31.19 (5.22)	27.35 (4.35)
Diabetes mellitus		
Yes	78 (4.4)	83 (4.7)
No	297 (16.9)	1,304 (74.0)
Hypertension		
Yes	348 (19.8)	307 (17.4)
No	27 (1.5)	1,080 (61.3)
Intake of beta-blockers		
Yes	219 (12.4)	184 (10.4)
No	156 (8.9)	1,203 (68.3)
Intake of ACE inhibitors		
Yes	178 (10.1)	104 (5.9)
No	197 (11.2)	1,283 (72.8)
Intake of statins		
Yes	124 (7.0)	153 (8.7)
No	251 (14.2)	1,234 (70.0)
Intake of fibrates		
Yes	3 (0.2)	8 (0.5)
No	372 (21.1)	1,379 (78.3)

*d*	Intake of statins Mean (SD) or n (%)	
	Yes (n = 277)	No (n = 1,485)
Age	65.66 (7.16)	59.92 (8.74)
Gender		
Male	161 (9.1)	695 (39.4)
Female	116 (6.6)	790 (44.8)
HDL-cholesterol (mmol/l)	1.37 (0.32)	1.47 (0.38)
LDL-cholesterol (mmol/l)	3.06 (0.76)	3.71 (0.89)
Total cholesterol (mmol/l)	5.14 (0.90)	5.83 (0.99)
Triglycerides (mmol/l)	1.69 (1.03)	1.47 (1.04)
BMI kg/m^2^	29.30 (4.85)	27.95 (4.77)
Diabetes mellitus		
Yes	60 (3.4)	101 (5.7)
No	217 (12.3)	1,384 (78.5)
Hypertension		
Yes	186 (10.6)	469 (26.6)
No	91 (5.2)	1,016 (57.7)
Intake of beta-blockers		
Yes	144 (8.2)	259 (14.7)
No	133 (7.5)	1,226 (69.6)
Intake of ACE inhibitors		
Yes	99 (5.6)	183 (10.4)
No	178 (10.1)	1,302 (73.9)
Intake of diuretics		
Yes	124 (7.0)	251 (14.2)
No	153 (8.7)	1,234 (70.0)
Intake of fibrates		
Yes	0 (0.0)	11 (0.6)
No	277 (15.7)	1,474 (83.7)

*e*	Intake of fibrates Mean (SD) or n (%)	
	Yes (n = 11)	No (n = 1,751)
Age	64.45 (8.99)	60.80 (8.761)
Gender		
Male	7 (0.4)	849 (48.2)
Female	4 (0.2)	902 (51.2)
HDL-cholesterol (mmol/l)	1.39 (0.58)	1.46 (0.38)
LDL-cholesterol (mmol/l)	3.55 (0.78)	3.62 (0.90)
Total cholesterol (mmol/l)	5.94 (0.70)	5.72 (1.01)
Triglycerides (mmol/l)	2.81 (2.82)	1.50 (1.02)
BMI kg/m^2^	28.40 (3.63)	28.16 (4.82)
Diabetes mellitus		
Yes	4 (0.2)	157 (8.9)
No	7 (0.4)	1,594 (90.5)
Hypertension		
Yes	7 (0.4)	648 (36.8)
No	4 (0.2)	1,103 (62.6)
Intake of beta-blockers		
Yes	5 (0.3)	398 (22.6)
No	6 (0.3)	1,353 (76.8)
Intake of ACE inhibitors		
Yes	3 (0.2)	279 (15.8)
No	8 (0.5)	1,472 (83.5)
Intake of diuretics		
Yes	3 (0.2)	372 (21.1)
No	8 (0.5)	1,379 (78.3)
Intake of statins		
Yes	0 (0.0)	277 (15.7)
No	11 (0.6)	1,474 (83.7)

## Materials and methods

### Study population

The research platform KORA conducts population-based surveys and subsequent follow-up studies in the fields of epidemiology, health care research, health economics and genetics. A multitude of different parameters is provided, including medical history, life style factors, and socio-demographic variables. The dataset analyzed here was taken from the F4 study, which was conducted in 2006–2008 as a follow-up of the fourth KORA survey (S4; 1999–2001). For this follow-up study F4, 3,080 of the 4,261 participants of the S4 were reexamined 7 years after baseline examination. From this group, the metabolic profiles of 1,768 participants, aged between 32 and 77 years were measured. In total, 1,762 of these study participants provided information about medication with beta-blockers, ACE inhibitors, diuretics, statins and/or fibrates. To determine the use of medication within the last 7 days before the examination an instrument for database-assisted online collection of medication data (IDOM) was applied [17]. The drug classes were considered according to the recommendations of the German Hypertension Association [18]. The characteristics of the population related to these drug classes are shown in Table 1a–e. The usage of the agents within each drug class is given in the *Supplemental Fig. 1*. Informed consent was obtained from each study participant and all study protocols were approved by the local ethics committees.

### Blood samples

For this study, we measured the metabolic profile in the blood serum of the study participants. To avoid variation due to circadian rhythm, blood was drawn in the morning between 8 and 10:30 am after overnight fasting (at least 8 h). Medication was taken in the morning as usual. Material was drawn into serum gel tubes, gently inverted twice and followed by 30 min resting at room temperature (18–25 °C) to obtain complete coagulation. The material was then centrifuged for 10 min (2750 g at 15 °C). Serum was aliquoted and stored at 4 °C, after which it was deep frozen to −80 °C on the same day until analysis of the metabolites.

### Metabolite profiling and metabolite spectrum

The metabolites were measured by the US-company Metabolon Inc., a commercial supplier of metabolic analyses. Their platform integrates the chemical analysis, including identification and relative quantification, data reduction, and quality assurance components of the process. Two separate ultrahigh performance liquid chromatography/tandem mass spectrometry (UHPLC/MS/MS; positive and negative mode) injections and one gas chromatography/mass spectrometry (GC/MS) injection were done on this platform.

The UHPLC/MS/MS platform utilized a Waters Acquity UPLC and a ThermoFisher LTQ mass spectrometer, while for GC/MS analysis a Thermo-Finnigan Trace DSQ MS was used.

A standard library containing retention time, molecular mass to charge ratio (m/z), preferred in-source fragments and adducts as well as their associated MS/MS spectra for all molecules in the library, subsequently enables to identify a multitude of metabolites in resulting MS/MS data. A more detailed description can be found in the Supplementary Material.

In a recent publication, Metabolon showed that their analytical platform is able to perform relative quantitative analysis of analytical data in a high-throughput mode and that it identifies a broad spectrum of molecules with a high degree of confidence [19]. Further details of the platform technology are described in Suhre et al. [20]. The measured panel includes 295 metabolites from many relevant classes such as amino acids, carbohydrates, cofactors and vitamins, acylcarnitines, glyceropospholipids, lipids, nucleotides, small peptides and xenobiotics.

This platform has been successfully used in several studies, e.g. for the analysis of the adult human plasma metabolome [21] and for the identification of sarcosine as a biomarker for prostate cancer [22].

### Statistical analysis

The statistical analysis system R (http://www.r-project.org/) and SPSS for Windows (Version 19.0. Chicago: SPSS Inc.) were used for the statistical analysis.

The analytic approach applied by Metabolon is semi-quantitative. This means that the added standards are mainly used for determining the retention time and not for the calculation of the metabolite concentrations. Thus, a relative intensity is measured and the measurement is sensitive to instrument parameters as well as fluctuations caused by maintenances like column change. These fluctuations are day dependent and the run day has to be considered for each individual. Thus, a run day normalization of the metabolic data was done: For each individual and each metabolite the data was first divided by the day median of the respective metabolite and then multiplied with the overall median of this metabolite.

To avoid false-positive associations, data points of metabolic traits that lay more than three standard deviations off the mean were excluded from further analysis. Metabolite concentrations were log-transformed since a test of normality showed that in most cases the log-transformed concentrations were closer to a normal distribution than the untransformed values.

For the identification of metabolites associated with medication use, a linear regression test was applied to model the association of each metabolite concentration and each of the five drug classes beta-blockers, ACE inhibitors, diuretics, statins and fibrates, respectively. As cofactors BMI, age, gender, HDL cholesterol, LDL cholesterol, total cholesterol, triglycerides, hypertension (>160/95 mmHg or known, medicated hypertension) and diabetes were chosen. To control for the effect of testing multiple hypotheses we used the Bonferroni correction. Thus, basing on a nominal level of 0.05, we used 3.39 × 10^−5^ [0.05/(295 metabolites * five drugs)] as an estimate of the significance level.

## Results

For each drug class—beta-blockers, ACE inhibitors, diuretics, statins and fibrates—we analyzed the metabolic dataset to identify metabolites whose blood serum levels significantly differ between subjects taking and not taking the respective drug (*p* value smaller than 3.39 × 10^−5^, the estimated significance level after correction for multiple testing—see “Statistical analysis”). The results of the linear regression are given in Table 2.

**Table 2:** Results of the linear regression test with the strongest association to beta-blockers, ACE inhibitors, diuretics, statins or fibrates

Medication	Metabolite	Pathway or metabolic class	Standard deviation/mean	*p* value	β^a^
Beta-blockers	Pyroglutamine	Glutamate metabolism	0.603	1.92 × 10^−9^	0.09
	Homocitrulline	Urea cycle; arginine, proline metabolism	0.661	9.62 × 10^−8^	0.07
	Serotonin	Tryptophan metabolism	0.387	3.11 × 10^−6^	−0.05
	Dihomolinoleate FA(20:2)	Essential fatty acid	0.394	4.49 × 10^−6^	−0.05
	Salicylate	Drug	3.714	4.98 × 10^−6^	0.36
	3-Hydroxybutyrate FA(4:0-OH)	Ketone bodies	1.274	6.65 × 10^−6^	−0.11
	10-Nonadecenoate FA(19:1)	Long chain fatty acid	0.381	6.70 × 10^−6^	−0.05
	Hydroxyisovaleroylcarnitine C4-OH-M	Valine, leucine and isoleucine metabolism	0.529	7.70 × 10^−6^	0.06
	Margarate FA(17:0)	Long chain fatty acid	0.302	1.67 × 10^−5^	−0.04
	Eicosenoate FA(20:1)	Long chain fatty acid	0.442	2.20 × 10^−5^	−0.05
	2-Methylbutyroylcarnitine C4-M	Valine, leucine and isoleucine metabolism	0.347	3.76 × 10^−5^	0.04
ACE inhibitors	Phenylalanylphenylalanine	Dipeptide	0.477	2.40 × 10^−80^	−0.27
	Aspartylphenylalanine	Dipeptide	0.667	2.07 × 10^−17^	−0.17
	HWESASXX	Polypeptide	1.290	4.16 × 10^−14^	0.13
	Bradykinin, des-arg(9)	Polypeptide	1.146	7.50 × 10^−13^	0.21
Diuretics	Pseudouridine	Pyrimidine metabolism, uracil containing	0.262	8.55 × 10^−10^	0.04
	C-glycosyltryptophan	Tryptophan metabolism	0.362	1.78 × 10^−8^	0.04
	Glutaroylcarnitine C5-DC	Lysine metabolism	0.380	2.75 × 10^−6^	0.05
	Phenylalanylphenylalanine	Dipeptide	0.477	1.19 × 10^−5^	−0.06
	Homocitrulline	Urea cycle; arginine, proline metabolism	0.661	1.62 × 10^−5^	0.06
	HWESASXX	Polypeptide	1.290	1.94 × 10^−5^	0.07
	Urate	Purine metabolism, urate metabolism	0.203	2.49 × 10^−5^	0.02
Statins	1-Arachidonoylglycerophosphocholine LPC(20:4)	Lysolipid	0.459	1.16 × 10^−12^	0.09
	1-Arachidonoylglycerophosphoethanolamine LPE(20:4)	Lysolipid	0.362	9.69 × 10^−10^	0.06
	7-Alpha-hydroxy-3-oxo-4-cholestenoate	Sterol/Steroid	0.326	2.76 × 10^−9^	−0.05
	1-Palmitoylglycerophosphoinositol LPI (16:0)	Lysolipid	0.495	1.95 × 10^−8^	−0.08
	Lathosterol	Sterol/Steroid	0.470	1.09 × 10^−7^	−0.12
	Isobutyrylcarnitine C3-M	Valine, leucine and isoleucine metabolism	0.518	5.27 × 10^−6^	0.06
	1-Docosahexaenoylglycerophosphocholine LPC (22:6)	Lysolipid	0.549	8.25 × 10^−6^	0.06
	Alpha-tocopherol	Tocopherol metabolism	0.311	1.63 × 10^−5^	0.04
	Glycochenodeoxycholate	Bile acid metabolism	3.288	1.98 × 10^−5^	−0.14
	Uridine	Pyrimidine metabolism, uracil containing	0.200	3,34 × 10^−5^	0.03
Fibrates	2-Hydroxyisobutyrate FA(3:0-OH-M)	Valine, leucine and isoleucine metabolism	0.701	1.01 × 10^−61^	0.72
	3-Dehydrocarnitine	Carnitine metabolism	0.317	9.78 × 10^−14^	0.29
	Riboflavin	Riboflavin metabolism	0.741	1.58 × 10^−11^	1.08
	Pantothenate	Pantothenate and CoA metabolism	0.474	1.30 × 10^−9^	0.27
	Indolelactate	Tryptophan metabolism	0.366	7.88 × 10^−8^	0.23
	Pyroglutamine	Glutamate metabolism	0.603	3.08 × 10^−7^	−0.32
	Carnitine	Carnitine metabolism	0.138	6.32 × 10^−7^	0.09
	Pipecolate	Lysine metabolism	0.893	5.14 × 10^−6^	0.27
	Uridine	Pyrimidine metabolism, uracil containing	0.201	1.87 × 10^−5^	0.12

Cofactors: BMI, age, gender, HDL cholesterol, LDL cholesterol, total cholesterol, triglycerides, hypertension and diabetes; the threshold for a significant *p* value is 3.39 × 10^−5^ [0.05/(295 metabolites × 5 drugs)]

*FA* fatty acid,* C* acyl carnitine,* OH* hydroxylic,* M* methylic,* DC* dicarboxylic,* LPC* lyso-glycerophosphocholine,* LPE* lyso-glycerophosphoethanolamine,* LPI* lyso-glycerophosphoinositol, (x:y) with x = chain length and y = double bonds

^a^Regression coefficient indicating the direction of the association (‘−’ negative association)

For patients who took beta-blockers we observed increased concentrations of pyroglutamine, homocitrulline, salicylate, and acylcarnitins in the blood serum. In contrast, serotonin, fatty acids and 3-hydroxybutyrate [FA(4:0-OH)] were decreased. Their *p* values ranged between 1.9 × 10^−9^ and 2.2 × 10^−5^.

For the group of ACE inhibitors we identified four metabolites that significantly associated with the intake of these drugs. The *p* values showed a broad range from 2.4 × 10^−80^ up to 7.5 × 10^−13^. While levels of HWESASXX and des-arg(9)-bradykinin were higher in case of medication with ACE inhibitors, we found lower levels of phenylalanylphenylalanine and aspartylphenylalanine. Diuretics showed associations with increased serum levels of pseudouridine, C-glycosyltryptophan, glutaroylcarnitine [C5-DC] and urate. Additional metabolites with a *p* value smaller than 3.39 × 10^−5^, namely homocitrulline, HWESASXX (both increased) and phenylalanylphenylalanine (decreased) were already found to associate with beta-blockers and ACE inhibitors, respectively.

To identify associations between lipid-lowering drugs and metabolites we analyzed the effects of statins as well as fibrates. For the statins the resulting metabolites with the lowest *p* values were 1-arachidonoylglycerophosphocholine [LPC(20:4)], 1-arachidonoylglycerophosphoethanolamine [LPE(20:4)], isobutyrylcarnitine [C3-M], 1-docosahexaenoylglycerophosphocholine [LPC(22:6)], alpha-tocopherol, uridine (all increased), 7-alphahydroxy-3-oxo-4-cholestenoate, 1-palmitoylglycerophosphoinositol [LPI(16:0)], lathosterol and glycochenodeoxycholate (all decreased). For fibrates most of the significant metabolites showed a positive association: 2-hydroxyisobutyrate [FA(3:0-OH-M)], 3-dehydrocarnitine, riboflavin, pantothenate, indolelactate, carnitine, pipecolate and uridine. Only for one of the resulting metabolites—pyroglutamine—a significant negative association was detected. Pyroglutamine was already observed to associate with the intake of beta-blockers. However, in contrast to the intake of fibrates, the association between the beta-blockers and the concentration of pyroglutamine was positive.

## Discussion

In this study we analyzed the effect of antihypertensive drugs and lipid-lowering drugs on the human metabolism. To this end, 295 metabolites were measured in the serum of 1,762 participants of the population-based KORA F4 study. We found hypothesis-generating associations with metabolites for four different drugs, however, not for diuretics. The results of the linear regression are given in Table 2. In the following we will discuss the main results for each drug class.

### Beta-blockers associate with decreased serotonin and free fatty acid levels

Beta-blockers diminish the effect of the sympathetic nervous system on its target organ mainly by inhibiting the action of noradrenaline and adrenaline on β-adrenergic receptors. Among the metabolites with the lowest *p* values we found several fatty acids that were decreased with beta-blocker intake and some acylcarnitines that were increased. The increase in the concentration of acylcarnitines is also supported by the only nominally significant results. The decrease of free fatty acids agrees with the function of beta-blockers inhibiting the action of noradrenaline and adrenaline on β-adrenergic receptors. Since lipolysis is dependent on the action of these hormones, less triglycerides are broken down to free fatty acids [23, 24]. Vanhees et al. [25] also reported this effect for the beta-blocker bisoprolol which reduced the availability of plasma free fatty acids in healthy men.

Serotonin, a monoamine neurotransmitter, was significantly decreased in the blood of participants taking beta-blockers. Low plasma levels of serotonin were found in depressive patients [26, 27] and depression has been discussed as side-effect of beta-blockers [28]. Hence, the serotonin level variation might explain an adverse effect.

The metabolite with the strongest association to the intake of beta-blockers was pyroglutamine—a cyclic derivative of glutamine—that showed an increased concentration. In this context, it is noteworthy that an opposite behaviour, namely a decrease in the pyroglutamine concentration was associated with fibrate intake. However, further research on this metabolite is needed since too little is known so far about its physiological role.

In summary, our results for beta-blockers show associations that may indicate side effects of this medication.

### Direct action of ACE inhibitors on its target ACE

The Angiotensin I-converting enzyme (ACE) cleaves not only angiotensin, but also several other polypeptides. One substrate of ACE is Cholecystokinin-8 (CCK-8) from which the dipeptide Asp-Phe-NH2 is cleaved [29]. In line with an inhibition of this process by ACE inhibitors, we found lower levels of aspartylphenylalanine (Asp-Phe) in users of ACE inhibitiors. Thus, one might expect that the substrate CCK-8 was increased in our patients on ACE inhibitors. Unfortunately, due to the used metabolite profiling technique CCK-8 was not in our metabolite panel and, therefore, we can only hypothesize. CCK-8 was shown to exert an antidiabetogenic action via increased insulin secretion [30]. Aguilar and Solomon suggested that ACE inhibitors may prevent the onset of diabetes [31] and Andraws and Brown could confirm this effect in a meta-analysis [32]. Thus, our observation of decreased levels of aspartylphenylalanine and the assumed elevated levels of CCK-8, suggest that this antidiabetogenic effect of ACE inhibitors is mediated by the inhibition of the cleavage of CCK-8. This hypothesis might be a starting point for further investigation.

The other dipeptide, phenylalanylphenylalanine, was also negatively associated with the intake of ACE inhibitors. It might be a product of the cleaving action of ACE as well, although the correspondent ACE substrate has not yet been identified. Candidate peptide substrates may now be searched for based on this information.

Another substrate of ACE is des-Arg(9)-bradykinin—the active metabolite of bradykinin—which causes blood vessels to dilate [33, 34]. Recent reports suggest an important role of lower bradykinin levels in the development of hypertension [35, 36]. Thus, our observation of an increased blood level of des-Arg(9)-bradykinin under ACE inhibitors indicates the direct action of this drug and strengthens the suggested role of bradykinin in the treatment with ACE inhibitors [37].

### Analysis of diuretics shows a heterogeneous picture

The results from the analysis of metabolic profiles of patients on diuretics are heterogeneous and difficult to interpret. An increased level of pseudouridine might indicate RNA degradation and cell turnover as well as the remodelling process in heart failure [38]. Our findings of an increase of C-glycosyltryptophan in patients on diuretics might be explained by the role of tryptophan. In liver cirrhosis the concentration of tryptophan is increased and tryptophan plays an important role in chronic kidney disease [39–41]. This is consistent with our observation of increased levels of glutaroylcarnitine [C5-DC], since glutaryl-CoA hydrogenase (GCDH) is essential in the degradation pathway of tryptophan [42]. A direct association between the metabolites pseudouridine and C-glycosyltryptophan and end stage renal disease (ESRD) was recently observed by Niewczas et al. [43]. They reported higher blood levels of these compounds in type 2 diabetes patients who progressed to ESRD within several years compared to diabetes patients without ESRD. Moreover, Yonemura et al. [44] observed a negative correlation between the C-glycosylated compound C-mannosyltryptophane and estimated glomerular filtration rate (eGFR; indicator of kidney function). An irreversible formation of homocitrulline (carbamoylation) is reported for end-stage renal disease and may explain the increased concentration of this metabolite [45].

Apart from their role as antihypertensives diuretics are used in cases of chronic kidney disease, heart failure and liver cirrhosis and, thus, we might see the metabolic traits of these diseases. Only the observed increased concentration of urate is a known side-effect of loop diuretics and thiazide [46].

Since more than 70 % of the participants on diuretics used the agent hydrochlorothiazide (HCT) we repeated the analysis only with HCT-users. The two resulting metabolites with significant associations with HCT are pseudouridine and C-glycosyltryptophan. These metabolites were also the top hits in the analysis of the diuretics which suggests that the results for diuretics mainly reflect the results for HCT.

### Statins and their effects on the synthesis and degradation of cholesterol

Statins lower the concentration of cholesterol by inhibiting the HMG-CoA-reductase (HMGCR) which is the rate controlling enzyme of the biosynthesis of cholesterol. The levels of lathosterol and 7-alpha-hydroxy-3-oxo-4-cholestenoate were decreased in the blood from study participants treated with statins. Lathosterol is a precursor of cholesterol in the cholesterol biosynthesis, while 7-alpha-hydroxy-3-oxo-4-cholestenoate is a metabolite of cholesterol [47, 48]. So these two metabolites show direct drug effects. The increased concentration of 1-arachidonoylglycerophosphocholine [LPC(20:4)] and 1-arachidonoylglycerophosphoethanolamine [LPE(20:4)] and the decreased level of 1-palmitoylglycerophosphoinositol [LPI(16:0)] agree with previous reports of an increase in arachidonic acid [FA(20:4)] synthesis and a decrease of palmitic acid [FA(16:0)] levels in persons on statins. This effect is assumed to be due to a statin-induced enhancement of delta five desaturase (FADS1) activity [49–51]. The benefit of the polyunsaturated fatty acid arachidonic acid is controversial. On the one hand arachidonic acid was reported as “beneficial in preventing and/or improving age-related declines in brain and cardiovascular system function” [52] as well as protective against oxidative stress in neurons [53]. On the other hand its role in inflammation is not fully clear, since anti- [54] as well as pro-inflammatory [55] effects have been described. A beneficial effect is achieved by the decrease of the 1-palmitoylglycerophosphoinositol [LPI(16:0)] carrying a saturated fatty acid which is considered as unhealthy.

In summary, for statins we observed mainly associations that are directly related to the action of this drug class.

### Fibrates: degradation marker and increased carnitine levels

As a second group of lipid-lowering drugs, we analyzed the intake of fibrates for associations with metabolic traits. Though the group size for fibrate intake was quite small, associations with small *p* values were observed due to the large effect size of the association. However, the possibility of a type I error cannot fully be excluded. The metabolite with the strongest association to fibrate intake was the upregulated 2-hydroxyisobutyrate [FA(3:0-OH-M)]. 2-hydroxyisobutyrate probably is a metabolite of fenofibrate. Liu et al. [56] propose a degradation of fenofibrate where reduced fenofibric acid is metabolized to reduced 4-chloro-4′-hydroxybenzophenone. We presume that 2-hydroxyisobutyrate is released from reduced fenofibric acid during this reaction. If this is correct, 2-hydroxyisobutyrate might be used as a marker for the degradation of fenofibrate. In addition, 2-hydroxyisobutyrate was reported to be associated with prediagnostic gestational diabetes mellitus conditions [57] and obesity, and it might also be derived from the gut microbiome [58].

We also observed associations that might be related to side-effects of fibrates. 3-Dehydrocarnitine and free carnitine showed an increased concentration in the blood of patients on fibrates. This agrees with previous findings that fenofibrate increases the level of carnitine [59, 60]. The fact that 3-dehydrocarnitine is an intermediate in the carnitine biosynthesis may explain the increase of the blood concentration of this metabolite. Pyroglutamine was already the result with the lowest *p* value in the beta-blockers analysis, but it also showed a strong association with the intake of fibrates. While with beta-blockers the association was positive, the association with fibrates was negative. The question if beta-blockers and fibrates influence the same metabolic phenomenon in opposite directions needs further investigation.

Our results show that indications for drug related metabolic changes can be identified in population studies although the participants are more influenced by their environment (e.g. xenobiotics, smoking, food, physical activity) and much less controlled than during a clinical drug testing, e.g. in phase 1. BMI, age, gender, HDL cholesterol, LDL cholesterol, total cholesterol, triglycerides, hypertension and diabetes are known to influence the here measured metabolic profile. To avoid seeing their disturbing influence in the results we used them as cofactors for our statistical analysis. In addition, we checked if any of the metabolites we found to be associated with beta-blockers, ACE inhibitors, statins or fibrates (not diuretics) shows an association with hypertension or the total blood cholesterol level in the non-medicated (no antihypertensives and no lipid-lowering drugs) participants. Except for one metabolite associated with the intake of statins (alpha-tocopherol), we found no such association, meaning that the associations observed for those drugs are mainly drug- and not disease-related. Also, a calculation considering for each drug the remaining four drugs as cofactors yielded the same significant associations.

### Limitations

Our sampling population was composed of Caucasians of European ancestry, so these results cannot be generalized to other ethnicities—effects of genetic variation on metabolism needs to be considered [20, 61, 62]. Because of the cross-sectional design of this study, we can only generate hypotheses based on associations—causal nature of the observed associations would require longitudinal data.

Major covariates, such as age, gender, BMI, HDL cholesterol, LDL cholesterol, total cholesterol, triglycerides, hypertension and diabetes were corrected for in the statistical analysis. However, in individual cases, interaction with other medication (e.g. with antiplatelet drugs or thyroid preparations) of the participants cannot be excluded.

To increase the statistical power the associations we reported in this paper were calculated for drug classes. It is not possible to trace the observed metabolic changes back to single pharmaceuticals. However, for each drug class specific associations could be clearly observed.

**Fig. 1:**
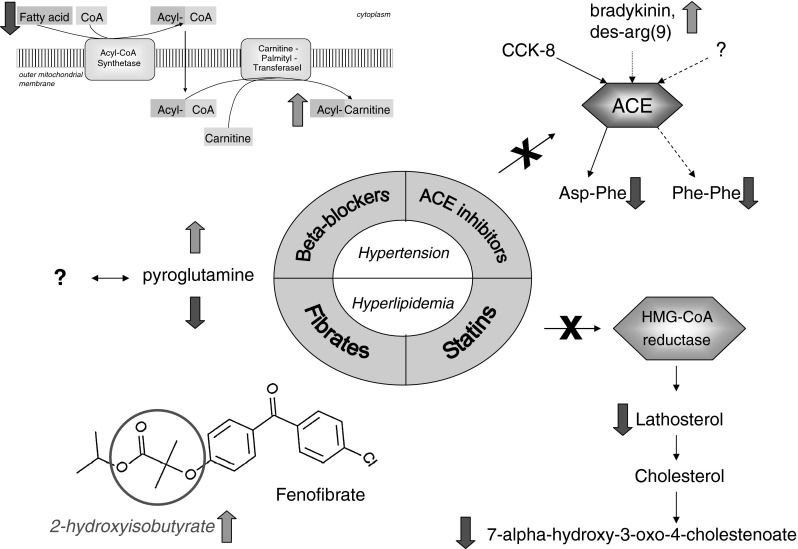
Summary of the main drug-metabolite associations. Beta-blockers associate with an impaired lipid metabolism; the action of ACE inhibitors can be seen from associations with changed levels of substrate and products of ACE; statins associate with metabolites involved in the biosynthesis and degradation of cholesterol; fibrates associate with a hypothesized breakdown product of fenofibrate; pyroglutamine shows contrariwise associations with fibrates and beta-blockers

In summary (Fig. 1), for beta-blockers we observed associations possibly related to side-effects, including a decreased concentration of free fatty acids and a possible relation to depression via a decreased serotonin level. The intake of ACE inhibitors and statins associated with metabolites that reflect the direct action of the agent on its target. For fibrates we observed a possible breakdown product. Taken together, these results now provide a starting point for further functional research on the action and side-effects of these drugs.

## Electronic supplementary material

Below is the link to the electronic supplementary material.

Supplementary material 1 (PDF 204 kb)
