# Supplementary material for: Metabolomics approach reveals effects of antihypertensives and lipid-lowering drugs on the human metabolism
Source: Eur J Epidemiol. 2014 May 10;29(5):325–36. doi: 10.1007/s10654-014-9910-7 (PMC4050296; doi:10.1007/s10654-014-9910-7)

Supplementary Material

Metabolomics approach reveals effects of antihypertensives and lipid-lowering drugs on the human metabolism

Journal:

European Journal of Epidemiology

Authors:

Elisabeth Altmaier, Gisela Fobo, Margit Heier, Barbara Thorand, Christine Meisinger, Werner Römisch-Margl, Melanie Waldenberger, Christian Gieger, Thomas Illig, Jerzy Adamski, Karsten Suhre, Gabi Kastenmüller

Corresponding author:

Elisabeth Altmaier
Helmholtz Zentrum München, German ResearchvCenter for Environmental Health,
Ingolstädter Landstr. 1,
D-85764 Neuherberg, Germany
e-mail: elisabeth.altmaier@helmholtz-muenchen.de

Metabolite profiling and metabolite spectrum

The metabolites were measured by the US-company Metabolon Inc., a commercial supplier of metabolic analyses. The platform they developed integrates the chemical analysis, including identification and relative quantification, data reduction, and quality assurance components of the process. For every sample proteins were precipitated and chemically diverse metabolites were isolated with methanol under vigorous shaking for 2 minutes followed by centrifugation. Two separate ultrahigh performance liquid chromatography/tandem mass spectrometry (UHPLC/MS/MS; positive and negative mode) injections and one gas chromatography/mass spectrometry (GC/MS) injection were done on this platform. In addition, one fraction of each sample was reserved for backup. Three types of controls were analyzed together with the KORA samples: extensively characterized human plasma samples, water samples and a cocktail of standards was spiked into every analyzed sample to allow instrument performance monitoring.

The UHPLC/MS/MS platform utilized a Waters Acquity UPLC and a ThermoFisher LTQ mass spectrometer, which included an electrospray ionization source and a linear ion-trap mass analyzer. For GC/MS analysis a Thermo-Finnigan Trace DSQ MS operated at unit mass resolving power with electron impact ionization and a 50-750 atomic mass unit scan range.

A standard library containing retention time, molecular mass to charge ratio (m/z), preferred in-source fragments and adducts as well as their associated MS/MS spectra for all molecules in the library, subsequently enables to identify a multitude of metabolites in resulting MS/MS data.

Supplemental Table 1. Metabolites measured in KORA F4.
For each metabolite we report the super-pathway, pathway and measurement platform. The biochemical identity of the metabolites is in general determined using adequate pure substances; in cases where metabolite identities were inferred based on their fragmentation spectrum and other biochemical evidence, these are indicated by a '*'.

metabolite	super-pathway	pathway	platform
alanine	Amino acid	Alanine and aspartate metabolism	GC/MS
asparagine	Amino acid	Alanine and aspartate metabolism	GC/MS
aspartate	Amino acid	Alanine and aspartate metabolism	GC/MS
N-acetylalanine	Amino acid	Alanine and aspartate metabolism	LC/MS neg
2-aminobutyrate	Amino acid	Butanoate metabolism	LC/MS pos
creatine	Amino acid	Creatine metabolism	LC/MS pos
creatinine	Amino acid	Creatine metabolism	LC/MS pos
2-hydroxybutyrate (AHB)	Amino acid	Cysteine, methionine, SAM, taurine metabolism	GC/MS
cysteine	Amino acid	Cysteine, methionine, SAM, taurine metabolism	GC/MS
cystine	Amino acid	Cysteine, methionine, SAM, taurine metabolism	GC/MS
methionine	Amino acid	Cysteine, methionine, SAM, taurine metabolism	LC/MS neg
glutamate	Amino acid	Glutamate metabolism	GC/MS
glutamine	Amino acid	Glutamate metabolism	LC/MS pos
pyroglutamine*	Amino acid	Glutamate metabolism	LC/MS pos
5-oxoproline	Amino acid	Glutathione metabolism	LC/MS pos
cysteine-glutathione disulfide	Amino acid	Glutathione metabolism	LC/MS pos
betaine	Amino acid	Glycine, serine and threonine metabolism	LC/MS pos
glycine	Amino acid	Glycine, serine and threonine metabolism	GC/MS
N-(2-furoyl)glycine	Amino acid	Glycine, serine and threonine metabolism	LC/MS pos
N-acetylglycine	Amino acid	Glycine, serine and threonine metabolism	GC/MS
N-acetylthreonine	Amino acid	Glycine, serine and threonine metabolism	LC/MS neg
serine	Amino acid	Glycine, serine and threonine metabolism	GC/MS
threonine	Amino acid	Glycine, serine and threonine metabolism	LC/MS pos

4-acetamidobutanoate	Amino acid	Guanidino and acetamido metabolism	LC/MS pos
3-methylhistidine	Amino acid	Histidine metabolism	LC/MS neg
histidine	Amino acid	Histidine metabolism	LC/MS neg
glutaroyl carnitine	Amino acid	Lysine metabolism	LC/MS pos
lysine	Amino acid	Lysine metabolism	LC/MS pos
pipecolate	Amino acid	Lysine metabolism	LC/MS pos
3-(3-hydroxyphenyl)propionate	Amino acid	Phenylalanine & tyrosine metabolism	LC/MS neg
3-(4-hydroxyphenyl)lactate	Amino acid	Phenylalanine & tyrosine metabolism	LC/MS neg
3-methoxytyrosine	Amino acid	Phenylalanine & tyrosine metabolism	LC/MS pos
3-phenylpropionate (hydrocinnamate)	Amino acid	Phenylalanine & tyrosine metabolism	LC/MS neg
4-hydroxyphenylacetate	Amino acid	Phenylalanine & tyrosine metabolism	GC/MS
p-cresol sulfate	Amino acid	Phenylalanine & tyrosine metabolism	LC/MS neg
phenol sulfate	Amino acid	Phenylalanine & tyrosine metabolism	LC/MS neg
phenylacetate	Amino acid	Phenylalanine & tyrosine metabolism	LC/MS neg
phenylacetylglutamine	Amino acid	Phenylalanine & tyrosine metabolism	LC/MS pos
phenylalanine	Amino acid	Phenylalanine & tyrosine metabolism	LC/MS pos
phenyllactate (PLA)	Amino acid	Phenylalanine & tyrosine metabolism	LC/MS neg
tyrosine	Amino acid	Phenylalanine & tyrosine metabolism	LC/MS pos
3-indoxyl sulfate	Amino acid	Tryptophan metabolism	LC/MS neg
C-glycosyltryptophan*	Amino acid	Tryptophan metabolism	LC/MS pos
indoleacetate	Amino acid	Tryptophan metabolism	LC/MS pos
indolelactate	Amino acid	Tryptophan metabolism	LC/MS pos
indolepropionate	Amino acid	Tryptophan metabolism	LC/MS pos
kynurenine	Amino acid	Tryptophan metabolism	LC/MS pos
serotonin (5HT)	Amino acid	Tryptophan metabolism	LC/MS pos

tryptophan	Amino acid	Tryptophan metabolism	LC/MS pos
arginine	Amino acid	Urea cycle; arginine-, proline-, metabolism	LC/MS neg
citrulline	Amino acid	Urea cycle; arginine-, proline-, metabolism	LC/MS pos
dimethylarginine (SDMA + ADMA)	Amino acid	Urea cycle; arginine-, proline-, metabolism	LC/MS pos
homocitrulline	Amino acid	Urea cycle; arginine-, proline-, metabolism	LC/MS pos
homostachydrine*	Amino acid	Urea cycle; arginine-, proline-, metabolism	LC/MS pos
N-acetylornithine	Amino acid	Urea cycle; arginine-, proline-, metabolism	LC/MS pos
ornithine	Amino acid	Urea cycle; arginine-, proline-, metabolism	LC/MS pos
proline	Amino acid	Urea cycle; arginine-, proline-, metabolism	LC/MS pos
stachydrine	Amino acid	Urea cycle; arginine-, proline-, metabolism	LC/MS pos
trans-4-hydroxyproline	Amino acid	Urea cycle; arginine-, proline-, metabolism	GC/MS
urea	Amino acid	Urea cycle; arginine-, proline-, metabolism	GC/MS
2-hydroxyisobutyrate	Amino acid	Valine, leucine and isoleucine metabolism	GC/MS
2-methylbutyroylcarnitine	Amino acid	Valine, leucine and isoleucine metabolism	LC/MS pos
3-hydroxy-2-ethylpropionate	Amino acid	Valine, leucine and isoleucine metabolism	GC/MS
3-methyl-2-oxobutyrate	Amino acid	Valine, leucine and isoleucine metabolism	LC/MS neg
3-methyl-2-oxovalerate	Amino acid	Valine, leucine and isoleucine metabolism	LC/MS neg
4-methyl-2-oxopentanoate	Amino acid	Valine, leucine and isoleucine metabolism	LC/MS neg
alpha-hydroxyisovalerate	Amino acid	Valine, leucine and isoleucine metabolism	LC/MS neg
beta-hydroxyisovalerate	Amino acid	Valine, leucine and isoleucine metabolism	LC/MS neg
hydroxyisovaleroyl carnitine	Amino acid	Valine, leucine and isoleucine metabolism	LC/MS pos
isobutyrylcarnitine	Amino acid	Valine, leucine and isoleucine metabolism	LC/MS pos
isoleucine	Amino acid	Valine, leucine and isoleucine metabolism	LC/MS pos
isovalerylcarnitine	Amino acid	Valine, leucine and isoleucine metabolism	LC/MS pos
leucine	Amino acid	Valine, leucine and isoleucine metabolism	LC/MS pos

levulinate (4-oxovalerate)	Amino acid	Valine, leucine and isoleucine metabolism	LC/MS pos or neg
tiglyl carnitine	Amino acid	Valine, leucine and isoleucine metabolism	LC/MS pos
valine	Amino acid	Valine, leucine and isoleucine metabolism	LC/MS pos
erythronate*	Carbohydrate	Aminosugars metabolism	GC/MS
erythrose	Carbohydrate	Fructose, mannose, galactose, starch, and sucrose metabolism	GC/MS
fructose	Carbohydrate	Fructose, mannose, galactose, starch, and sucrose metabolism	GC/MS
mannitol	Carbohydrate	Fructose, mannose, galactose, starch, and sucrose metabolism	GC/MS
mannose	Carbohydrate	Fructose, mannose, galactose, starch, and sucrose metabolism	GC/MS
1,5-anhydroglucitol (1,5-AG)	Carbohydrate	Glycolysis, gluconeogenesis, pyruvate metabolism	LC/MS neg
1,6-anhydroglucose	Carbohydrate	Glycolysis, gluconeogenesis, pyruvate metabolism	GC/MS
glucose	Carbohydrate	Glycolysis, gluconeogenesis, pyruvate metabolism	GC/MS
glycerate	Carbohydrate	Glycolysis, gluconeogenesis, pyruvate metabolism	GC/MS
lactate	Carbohydrate	Glycolysis, gluconeogenesis, pyruvate metabolism	GC/MS
pyruvate	Carbohydrate	Glycolysis, gluconeogenesis, pyruvate metabolism	GC/MS
arabinose	Carbohydrate	Nucleotide sugars, pentose metabolism	GC/MS
arabitol	Carbohydrate	Nucleotide sugars, pentose metabolism	GC/MS
threitol	Carbohydrate	Nucleotide sugars, pentose metabolism	GC/MS
ascorbate (Vitamin C)	Cofactors and vitamins	Ascorbate and aldarate metabolism	GC/MS
threonate	Cofactors and vitamins	Ascorbate and aldarate metabolism	GC/MS
heme*	Cofactors and vitamins	Hemoglobin and porphyrin	LC/MS pos

bilirubin (E,E)*	Cofactors and vitamins	Hemoglobin and porphyrin metabolism	LC/MS pos
bilirubin (E,Z or Z,E)*	Cofactors and vitamins	Hemoglobin and porphyrin metabolism	LC/MS pos
bilirubin (Z,Z)	Cofactors and vitamins	Hemoglobin and porphyrin metabolism	LC/MS neg
biliverdin	Cofactors and vitamins	Hemoglobin and porphyrin metabolism	LC/MS neg
trigonelline (N'-methylnicotinate)	Cofactors and vitamins	Nicotinate and nicotinamide metabolism	LC/MS pos
pantothenate	Cofactors and vitamins	Pantothenate and CoA metabolism	LC/MS pos
riboflavin (Vitamin B2)	Cofactors and vitamins	Riboflavin metabolism	LC/MS pos
alpha-tocopherol	Cofactors and vitamins	Tocopherol metabolism	GC/MS
gamma-tocopherol	Cofactors and vitamins	Tocopherol metabolism	GC/MS
pyridoxate	Cofactors and vitamins	Vitamin B6 metabolism	LC/MS neg
alpha-ketoglutarate	Energy	Krebs cycle	GC/MS
citrate	Energy	Krebs cycle	GC/MS
malate	Energy	Krebs cycle	GC/MS
succinylcarnitine	Energy	Krebs cycle	LC/MS pos
acetylphosphate	Energy	Oxidative phosphorylation	GC/MS
phosphate	Energy	Oxidative phosphorylation	GC/MS
cholate	Lipid	Bile acid metabolism	LC/MS neg
deoxycholate	Lipid	Bile acid metabolism	LC/MS neg
glycochenodeoxycholate	Lipid	Bile acid metabolism	LC/MS neg
glycocholate	Lipid	Bile acid metabolism	LC/MS pos
glycodeoxycholate	Lipid	Bile acid metabolism	LC/MS neg
hyodeoxycholate	Lipid	Bile acid metabolism	LC/MS neg
taurochenodeoxycholate	Lipid	Bile acid metabolism	LC/MS neg
taurocholate	Lipid	Bile acid metabolism	LC/MS neg
taurodeoxycholate	Lipid	Bile acid metabolism	LC/MS neg

taurolithocholate 3-sulfate	Lipid	Bile acid metabolism	LC/MS neg
ursodeoxycholate	Lipid	Bile acid metabolism	LC/MS neg
2-tetradecenoyl carnitine	Lipid	Carnitine metabolism	LC/MS pos
3-dehydrocarnitine*	Lipid	Carnitine metabolism	LC/MS pos
acetylcarnitine	Lipid	Carnitine metabolism	LC/MS pos
carnitine	Lipid	Carnitine metabolism	LC/MS pos
decanoylcarnitine	Lipid	Carnitine metabolism	LC/MS pos
hexanoylcarnitine	Lipid	Carnitine metabolism	LC/MS pos
laurylcarnitine	Lipid	Carnitine metabolism	LC/MS pos
octanoylcarnitine	Lipid	Carnitine metabolism	LC/MS pos
oleoylcarnitine	Lipid	Carnitine metabolism	LC/MS pos
palmitoylcarnitine	Lipid	Carnitine metabolism	LC/MS pos
stearoylcarnitine	Lipid	Carnitine metabolism	LC/MS pos
thromboxane B2	Lipid	Eicosanoid	LC/MS neg
dihomo-linolenate (20:3n3 or n6)	Lipid	Essential fatty acid	LC/MS neg
docosahexaenoate (DHA; 22:6n3)	Lipid	Essential fatty acid	LC/MS neg
docosapentaenoate (n3 DPA; 22:5n3)	Lipid	Essential fatty acid	LC/MS neg
eicosapentaenoate (EPA; 20:5n3)	Lipid	Essential fatty acid	LC/MS neg
linolenate [alpha or gamma; (18:3n3 or 6)]	Lipid	Essential fatty acid	LC/MS neg
isovalerate	Lipid	Fatty acid metabolism	LC/MS neg
butyrylcarnitine	Lipid	Fatty acid metabolism (also BCAA metabolism)	LC/MS pos
propionylcarnitine	Lipid	Fatty acid metabolism (also BCAA metabolism)	LC/MS pos
linoleamide (18:2n6)	Lipid	Fatty acid, amide	LC/MS pos
oleamide	Lipid	Fatty acid, amide	LC/MS pos
3-carboxy-4-methyl-5-propyl-2-furanpropanoate (CMPF)	Lipid	Fatty acid, dicarboxylate	LC/MS neg

dodecanedioate	Lipid	Fatty acid, dicarboxylate	LC/MS neg
hexadecanedioate	Lipid	Fatty acid, dicarboxylate	LC/MS neg
octadecanedioate	Lipid	Fatty acid, dicarboxylate	LC/MS neg
sebacate (decanedioate)	Lipid	Fatty acid, dicarboxylate	LC/MS neg
tetradecanedioate	Lipid	Fatty acid, dicarboxylate	LC/MS neg
n-Butyl Oleate	Lipid	Fatty acid, ester	GC/MS
2-hydroxypalmitate	Lipid	Fatty acid, monohydroxy	LC/MS neg
2-hydroxystearate	Lipid	Fatty acid, monohydroxy	LC/MS neg
choline	Lipid	Glycerolipid metabolism	LC/MS pos
glycerol	Lipid	Glycerolipid metabolism	GC/MS
glycerol 3-phosphate (G3P)	Lipid	Glycerolipid metabolism	GC/MS
glycerophosphorylcholine (GPC)	Lipid	Glycerolipid metabolism	LC/MS pos
inositol 1-phosphate (I1P)	Lipid	Inositol metabolism	GC/MS
myo-inositol	Lipid	Inositol metabolism	GC/MS
scyllo-inositol	Lipid	Inositol metabolism	GC/MS
3-hydroxybutyrate (BHBA)	Lipid	Ketone bodies	GC/MS
10-heptadecenoate (17:1n7)	Lipid	Long chain fatty acid	LC/MS neg
10-nonadecenoate (19:1n9)	Lipid	Long chain fatty acid	LC/MS neg
adrenate (22:4n6)	Lipid	Long chain fatty acid	LC/MS neg
arachidonate (20:4n6)	Lipid	Long chain fatty acid	LC/MS neg
dihomo-linoleate (20:2n6)	Lipid	Long chain fatty acid	LC/MS neg
eicosenoate (20:1n9 or 11)	Lipid	Long chain fatty acid	LC/MS neg
linoleate (18:2n6)	Lipid	Long chain fatty acid	LC/MS neg
margarate (17:0)	Lipid	Long chain fatty acid	LC/MS neg
myristate (14:0)	Lipid	Long chain fatty acid	LC/MS neg

myristoleate (14:1n5)	Lipid	Long chain fatty acid	LC/MS neg
nonadecanoate (19:0)	Lipid	Long chain fatty acid	LC/MS neg
oleate (18:1n9)	Lipid	Long chain fatty acid	LC/MS neg
palmitate (16:0)	Lipid	Long chain fatty acid	LC/MS neg
palmitoleate (16:1n7)	Lipid	Long chain fatty acid	LC/MS neg
pentadecanoate (15:0)	Lipid	Long chain fatty acid	GC/MS
stearate (18:0)	Lipid	Long chain fatty acid	LC/MS neg
stearidonate (18:4n3)	Lipid	Long chain fatty acid	LC/MS neg
1-arachidonoylglycerophosphocholine*	Lipid	Lysolipid	LC/MS pos
1-arachidonoylglycerophosphoethanolamine*	Lipid	Lysolipid	LC/MS neg
1-arachidonoylglycerophosphoinositol*	Lipid	Lysolipid	LC/MS neg
1-docosahexaenoylglycerophosphocholine*	Lipid	Lysolipid	LC/MS pos
1-eicosadienoylglycerophosphocholine*	Lipid	Lysolipid	LC/MS pos
1-eicosatrienoylglycerophosphocholine*	Lipid	Lysolipid	LC/MS pos
1-heptadecanoylglycerophosphocholine	Lipid	Lysolipid	LC/MS pos
1-linoleoylglycerophosphocholine	Lipid	Lysolipid	LC/MS pos
1-linoleoylglycerophosphoethanolamine*	Lipid	Lysolipid	LC/MS neg
1-myristoylglycerophosphocholine	Lipid	Lysolipid	LC/MS pos
1-oleoylglycerophosphocholine	Lipid	Lysolipid	LC/MS pos
1-oleoylglycerophosphoethanolamine	Lipid	Lysolipid	LC/MS neg
1-palmitoleoylglycerophosphocholine*	Lipid	Lysolipid	LC/MS pos
1-palmitoylglycerophosphocholine	Lipid	Lysolipid	LC/MS pos
1-palmitoylglycerophosphoethanolamine	Lipid	Lysolipid	LC/MS neg
1-palmitoylglycerophosphoinositol*	Lipid	Lysolipid	LC/MS neg
1-stearoylglycerophosphocholine	Lipid	Lysolipid	LC/MS pos

1-stearoylglycerophosphoethanolamine	Lipid	Lysolipid	LC/MS neg
1-stearoylglycerophosphoinositol	Lipid	Lysolipid	LC/MS neg
2-linoleoylglycerophosphocholine*	Lipid	Lysolipid	LC/MS pos
2-linoleoylglycerophosphoethanolamine*	Lipid	Lysolipid	LC/MS neg
2-oleoylglycerophosphocholine*	Lipid	Lysolipid	LC/MS pos
2-palmitoylglycerophosphocholine*	Lipid	Lysolipid	LC/MS pos
2-stearoylglycerophosphocholine*	Lipid	Lysolipid	LC/MS pos
10-undecenoate (11:1n1)	Lipid	Medium chain fatty acid	LC/MS neg
5-dodecenoate (12:1n7)	Lipid	Medium chain fatty acid	LC/MS neg
caprate (10:0)	Lipid	Medium chain fatty acid	LC/MS neg
caproate (6:0)	Lipid	Medium chain fatty acid	LC/MS neg
caprylate (8:0)	Lipid	Medium chain fatty acid	LC/MS neg
heptanoate (7:0)	Lipid	Medium chain fatty acid	LC/MS neg
laurate (12:0)	Lipid	Medium chain fatty acid	LC/MS neg
pelargonate (9:0)	Lipid	Medium chain fatty acid	LC/MS neg
undecanoate (11:0)	Lipid	Medium chain fatty acid	LC/MS neg
1-linoleoylglycerol (1-monolinolein)	Lipid	Monoacylglycerol	LC/MS neg
1-oleoylglycerol (1-monoolein)	Lipid	Monoacylglycerol	LC/MS pos
1-palmitoylglycerol (1-monopalmitin)	Lipid	Monoacylglycerol	GC/MS
1-stearoylglycerol (1-monostearin)	Lipid	Monoacylglycerol	GC/MS
valerate	Lipid	Short chain fatty acid	LC/MS neg
7-alpha-hydroxy-3-oxo-4-cholestenoate (7-Hoca)	Lipid	Sterol/Steroid	LC/MS neg
androsterone sulfate	Lipid	Sterol/Steroid	LC/MS neg
cholesterol	Lipid	Sterol/Steroid	GC/MS
cortisol	Lipid	Sterol/Steroid	LC/MS pos

cortisone	Lipid	Sterol/Steroid	LC/MS pos
dehydroisoandrosterone sulfate (DHEA-S)	Lipid	Sterol/Steroid	LC/MS neg
epiandrosterone sulfate	Lipid	Sterol/Steroid	LC/MS neg
estrone 3-sulfate	Lipid	Sterol/Steroid	LC/MS neg
lathosterol	Lipid	Sterol/Steroid	GC/MS
carbamazepine*			LC/MS pos
hypoxanthine	Nucleotide	Purine metabolism, (hypo)xanthine/inosine containing	LC/MS neg
inosine	Nucleotide	Purine metabolism, (hypo)xanthine/inosine containing	LC/MS neg
xanthine	Nucleotide	Purine metabolism, (hypo)xanthine/inosine containing	LC/MS pos
adenosine	Nucleotide	Purine metabolism, adenine containing	LC/MS pos
N1-methyladenosine	Nucleotide	Purine metabolism, adenine containing	LC/MS pos
7-methylguanine	Nucleotide	Purine metabolism, guanine containing	LC/MS pos
guanosine	Nucleotide	Purine metabolism, guanine containing	LC/MS pos
N2,N2-dimethylguanosine	Nucleotide	Purine metabolism, guanine containing	LC/MS pos
allantoin	Nucleotide	Purine metabolism, urate metabolism	GC/MS
urate	Nucleotide	Purine metabolism, urate metabolism	LC/MS neg
pseudouridine	Nucleotide	Pyrimidine metabolism, uracil containing	LC/MS pos
uridine	Nucleotide	Pyrimidine metabolism, uracil containing	LC/MS neg
aspartylphenylalanine	Peptide	Dipeptide	LC/MS pos
glycylvaline	Peptide	Dipeptide	LC/MS pos
leucylleucine	Peptide	Dipeptide	LC/MS pos
phenylalanylphenylalanine	Peptide	Dipeptide	LC/MS pos
pro-hydroxy-pro	Peptide	Dipeptide	LC/MS pos

pyroglutamylglycine	Peptide	Dipeptide	LC/MS neg
ADpSGEGDFXAEGGGVR*	Peptide	Fibrinogen cleavage peptide	LC/MS pos
ADSGEGDFXAEGGGVR*	Peptide	Fibrinogen cleavage peptide	LC/MS pos
DSGEGDFXAEGGGVR*	Peptide	Fibrinogen cleavage peptide	LC/MS pos
gamma-glutamylglutamate	Peptide	gamma-glutamyl	LC/MS pos
gamma-glutamylglutamine	Peptide	gamma-glutamyl	LC/MS pos
gamma-glutamylisoleucine*	Peptide	gamma-glutamyl	LC/MS pos
gamma-glutamylleucine	Peptide	gamma-glutamyl	LC/MS pos
gamma-glutamylmethionine*	Peptide	gamma-glutamyl	LC/MS pos
gamma-glutamylphenylalanine	Peptide	gamma-glutamyl	LC/MS pos
gamma-glutamylthreonine*	Peptide	gamma-glutamyl	LC/MS pos
gamma-glutamyltyrosine	Peptide	gamma-glutamyl	LC/MS pos
gamma-glutamylvaline	Peptide	gamma-glutamyl	LC/MS pos
bradykinin, des-arg(9)	Peptide	Polypeptide	LC/MS pos
HWESASXX*	Peptide	Polypeptide	LC/MS pos
2-hydroxyhippurate (salicylurate)	Xenobiotics	Benzoate metabolism	LC/MS neg
3-ethylphenylsulfate*	Xenobiotics	Benzoate metabolism	LC/MS neg
4-ethylphenylsulfate	Xenobiotics	Benzoate metabolism	LC/MS neg
4-vinylphenol sulfate	Xenobiotics	Benzoate metabolism	LC/MS neg
benzoate	Xenobiotics	Benzoate metabolism	LC/MS neg
catechol sulfate	Xenobiotics	Benzoate metabolism	LC/MS neg
hippurate	Xenobiotics	Benzoate metabolism	LC/MS pos
glycerol 2-phosphate	Xenobiotics	Chemical	GC/MS
2-hydroxyacetaminophen sulfate*	Xenobiotics	Drug	LC/MS neg
2-methoxyacetaminophen sulfate*	Xenobiotics	Drug	LC/MS neg

3-(cystein-S-yl)acetaminophen*	Xenobiotics	Drug	LC/MS pos
4-acetamidophenol	Xenobiotics	Drug	GC/MS
4-acetaminophen sulfate	Xenobiotics	Drug	LC/MS neg
hydroquinone sulfate	Xenobiotics	Drug	LC/MS neg
hydroxypioglitazone*	Xenobiotics	Drug	LC/MS pos
ibuprofen	Xenobiotics	Drug	LC/MS neg
metoprolol	Xenobiotics	Drug	LC/MS pos
metoprolol acid metabolite*	Xenobiotics	Drug	LC/MS pos
naproxen	Xenobiotics	Drug	LC/MS neg
p-acetamidophenylglucuronide	Xenobiotics	Drug	LC/MS pos
pioglitazone*	Xenobiotics	Drug	LC/MS pos
salicylate	Xenobiotics	Drug	GC/MS
salicyluric glucuronide*	Xenobiotics	Drug	LC/MS neg
piperine	Xenobiotics	Food component/Plant	LC/MS pos
quinate	Xenobiotics	Food component/Plant	GC/MS
saccharin	Xenobiotics	Food component/Plant	LC/MS neg
thymol sulfate	Xenobiotics	Food component/Plant	LC/MS neg
erythritol	Xenobiotics	Sugar, sugar substitute, starch	GC/MS
cotinine	Xenobiotics	Tobacco metabolite	LC/MS pos
1,3,7-trimethylurate	Xenobiotics	Xanthine metabolism	LC/MS neg
1,7-dimethylurate	Xenobiotics	Xanthine metabolism	LC/MS neg
1-methylurate	Xenobiotics	Xanthine metabolism	LC/MS pos
1-methylxanthine	Xenobiotics	Xanthine metabolism	LC/MS pos
3-methylxanthine	Xenobiotics	Xanthine metabolism	LC/MS pos
7-methylxanthine	Xenobiotics	Xanthine metabolism	LC/MS pos

caffeine	Xenobiotics	Xanthine metabolism	LC/MS pos
paraxanthine	Xenobiotics	Xanthine metabolism	LC/MS pos
theobromine	Xenobiotics	Xanthine metabolism	LC/MS pos
theophylline	Xenobiotics	Xanthine metabolism	LC/MS neg

Supplemental Fig. 1: Distribution of the agents for each drug class in %

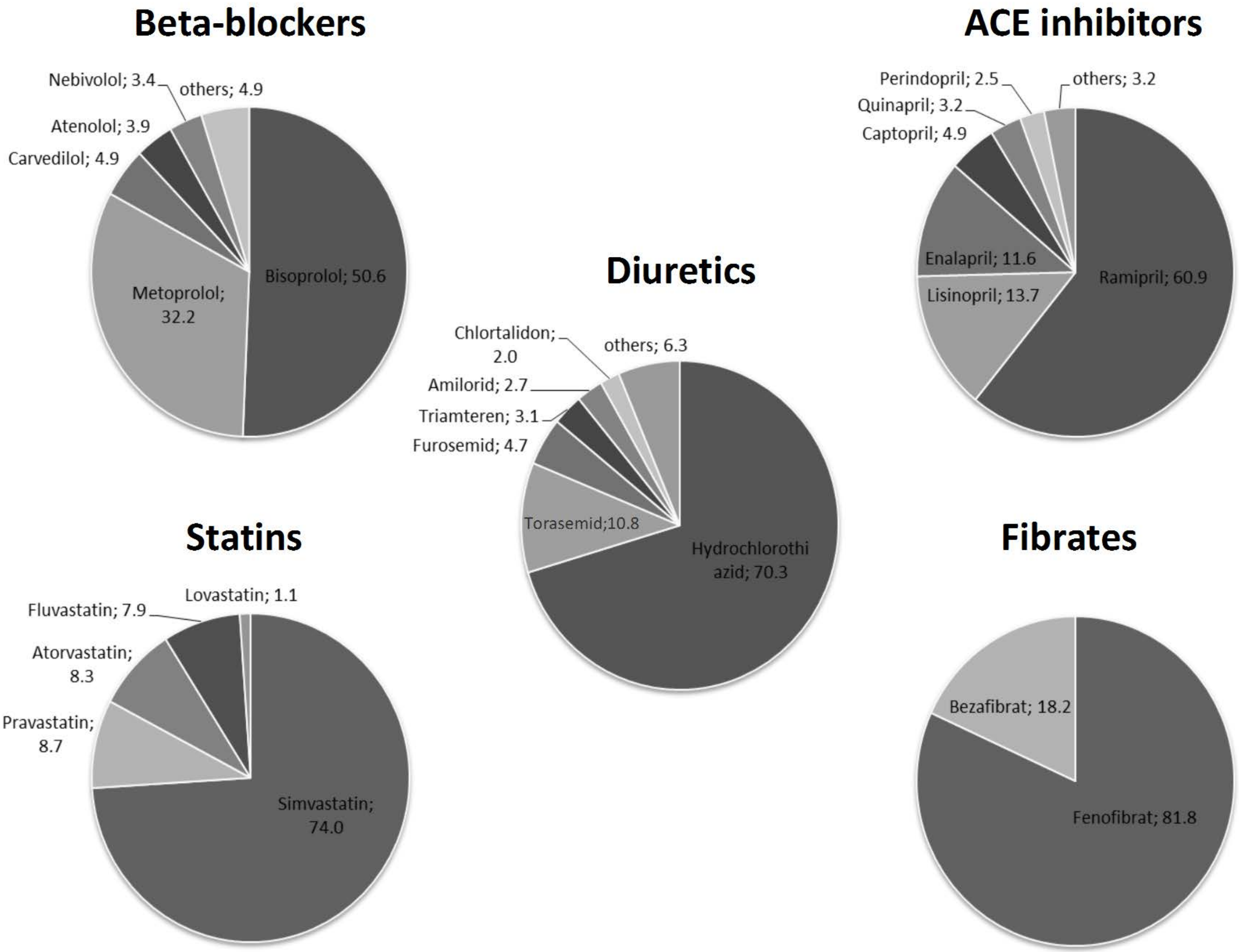

Supplement: Supplementary file 1 — Supplementary material 1 (PDF 204 kb) [file 10654_2014_9910_MOESM1_ESM.pdf]
